# Critical classification parameters linking species to Plant Functional Type in African ecosystems

**DOI:** 10.1038/s41597-026-06728-z

**Published:** 2026-02-03

**Authors:** Enimhien F. Akhabue, Andrew M. Cunliffe, Karina Bett-Williams, Anna B. Harper, Petra Holden, Tom Powell

**Affiliations:** 1 Department of Geography, University of Exeter, Exeter, United Kingdom; 2 Global Systems Institute, University of Exeter, Exeter, United Kingdom; 3 UK Meteorological Office, Exeter, United Kingdom; 4 University of Georgia, Athens, Georgia United States of America; 5 African Climate & Development Initiative, University of Cape Town, Cape Town, South Africa

**Keywords:** Plant ecology, Ecological modelling

## Abstract

Accurately representing African ecosystems in land surface models (LSMs) remains challenging due to the limited availability and accessibility of ecological data like plant traits. We systematically classified African plant species represented in the TRY plant trait database into Plant Functional Types (PFTs) consistent with those in the JULES LSM, to enable improvements of PFT parameterization in these models. From the TRY database plant trait observations were obtained representing 2,082 plant species. We assigned classification parameters including growth form, leaf type, leaf phenology, photosynthetic pathway and climate zone using multiple sources. This delivered a sixfold increase in number of plant species that could be mapped to PFT classes from 265 to 1603 representing 137 families. It delivered a fivefold increase in the number of useable observations among the 27 traits evaluated. Our lookup table can be used to integrate existing plant trait data into PFT parameterisations in land surface models and similar large scale modelling exercises, to enhance the representation of African ecosystems and improve their capacity to simulate African ecosystems.

## Background & Summary

The accurate representation of African plant species traits within the parameterization of Plant Functional Types (PFTs) in land surface models (LSM) is important for improving modelled predictions of environmental change. Despite Africa’s exceptional biodiversity and ecological importance, its ecosystems remain underrepresented in most global models, which still fail to capture their full complexity and diversity. This limitation is largely driven by a lack of scientific attention and data limitations, arising from the understudy of African biodiversity, the unique nature of complex African ecosystems, and the gap in continuous, high-quality datasets^1,2^. Although databases such as the TRY plants traits database (try-db.org) provide an invaluable foundation for global modelling studies, their geographic coverage is uneven, with significant underrepresentation of most African regions^3,4^. These longstanding data biases can hinder the effective application of PFTs in modelling efforts and underscores the need for continued data collection and collation in underrepresented areas.

PFTs classify plant species into various groups based on their functional characteristics in an ecosystem^5–7^. This approach helps simplify the complex nature of plant diversity for representation in LSMs like the Joint UK Land Environment Simulator (JULES) and other coupled climate models^8–11^. PFTs are important for modelling ecosystem processes, vegetation dynamics, carbon dynamics, and climate change. In LSMs, vegetation is represented by PFTs that are often treated as separate sub-grid tiles (area fractions) within each model grid box^12^. The model computes energy, water, and carbon exchanges for each tile separately and then aggregates them to the grid-box level. Each PFT is defined by a set of parameters that describe various aspects of their physiology, radiative properties, seasonal responses, and other characteristics. These parameters provide a simplified description of how different types of plants function and interact with their environment^13^. The relationships between functional traits and environmental factors are important for understanding ecosystem functioning^14^, as environmental heterogeneity can drive trait variation in ecosystems^15–18^. While substantial data exist for many regions such as Europe and North America, regions such as tropical Africa as well as Central/West Asia, and the Middle East remain underrepresented in key ecological datasets, with both species interactions and trait records poorly documented^14,19,20^.

JULES is a widely used land surface model that simulates carbon, water, and energy exchanges between the land and atmosphere, contributing to global climate projections. It forms the land surface component of the UK Earth System Model, contributes to Coupled Model Intercomparison Project (CMIP) analyses, and supports international initiatives such as the Global Carbon Budget and the TRENDY intercomparison project^21–23^. Its central role in land surface and climate research is reflected in its use in more than 317 publications in the last 25 years (based on a Europe PMC query using the *europepmc* R package), underscoring its importance for modelling terrestrial fluxes and global climate processes. JULES’s ability to represent vegetation dynamics is crucial for modelling terrestrial ecosystems. Originally, it classified vegetation into five PFTs^24^. In 2016, trait data from TRY enabled new trait-based parameterisations that distinguished deciduous from evergreen vegetation, increasing the number of PFTs from five to nine^10^ (Table 1). This refinement improved simulations of vegetation distribution, gross primary productivity (GPP), net primary productivity (NPP), and carbon fluxes relative to the original classification^25^.

**Table 1:** JULES currently represents plants through nine Plant Functional Types (PFTs).

Tropical broadleaf evergreen trees (BET-Tr)
Temperate broadleaf evergreen trees (BET-Te)
Broadleaf deciduous trees (BDT)
Needleleaf evergreen trees (NET)
Needleleaf deciduous trees (NDT)
C3 grass
C4 grass
Evergreen shrubs (ESh)
Deciduous shrubs (DSh)

Improving the availability and use of plant trait observations is essential for understanding Africa’s diverse ecosystems and enhancing their representations in LSMs. In this paper, we present a harmonised dataset that systematically maps African plant species into model-relevant PFTs to redress key regional data gaps. Our approach aligns with the JULES PFT framework and can be translated to other PFT schemes, including those used in ORCHIDEE (Organising Carbon and Hydrology in Dynamic Ecosystems), LPJ-GUESS (Lund-Postdam-Jena General Ecosystem Simulator), and CLM (Community Land Model)^26–28^. This resource substantially increases the availability of floral traits data to parameterise and evaluate land surface modelling frameworks. It is especially valuable for African landscapes, which remain underrepresented in global collections of scientific information^29^, and exhibit functional characteristics, such as fire adaptation, that differ from those of the temperate ecosystems on which many modelling frameworks are based. The Africa-specific PFT coverage enables the derivation of more regionally appropriate model parameters improving model simulations.

## Methods

### Description of the TRY database and pre-processing of data

The current version of the TRY database contains plant traits data - morphological, anatomical, physiological, biochemical and phenological characteristics of 15.4 million trait records across 2,661 traits for 305,600 plant taxa, based on measurements of over six million individual plants and their component organs and/or tissues^20,30^. This information underpins key understanding of ecosystems and their capacity to adapt to global change and biodiversity loss. Datasets for 27 plant traits (see the full list in the codebase linked in the code availability section) were requested from the TRY database, and the data releases associated with these requests were processed on 6 December 2023, 7 May 2024, and 27 August 2024. Only records available up to these dates were included in the analysis. Our study primarily focused on unrestricted observations (data openly accessible without special permission) within the TRY database. In some cases, data contributors granted additional access to restricted datasets.

### Geographic filtering

All analysis including the pre-processing and trait-based classification were conducted in R v4.1.2^31^. The full codebase for the analysis and resulting outputs are available at 10.5281/zenodo.16533069. From the TRY database we retrieved 666,730 trait records (a ‘trait record’ is a single trait measurement with associated metadata). After removing records with zero trait values and unnamed species, 663,173 records remained. Using the *sf* package (v1.0.19), we overlaid record coordinates with Natural Earth Admin 0 country polygons (v1.0.1) to select records within Africa’s geographic extent. This spatial filter retained 47,688 records (7.19%) which formed the African subset. The cleaned global TRY dataset spans 22,108 accepted plant species, of which 2,084 (9.4%) occur in the Africa subset. All subsequent classification steps use the Africa subset.

### Taxonomic harmonization

We conducted taxonomic harmonization of species names using the *WorldFlora* R package (v1.14.5)^32^, which standardizes botanical names based on the World Flora Online (WFO) taxonomic backbone^33^. The harmonization process began by preprocessing species names using the WFO.prepare() function to remove potential author citations, punctuation, and infraspecific markers, ensuring that all names were in a consistent binomial format. Cleaned names were matched against the WFO backbone using WFO.match() with default settings, and the best match for each name was retained using WFO.one(). The final harmonized dataset included the original species name, the matched WFO name, taxonomic status (e.g., accepted, synonym), and accepted name where applicable.

**Fig. 1:**
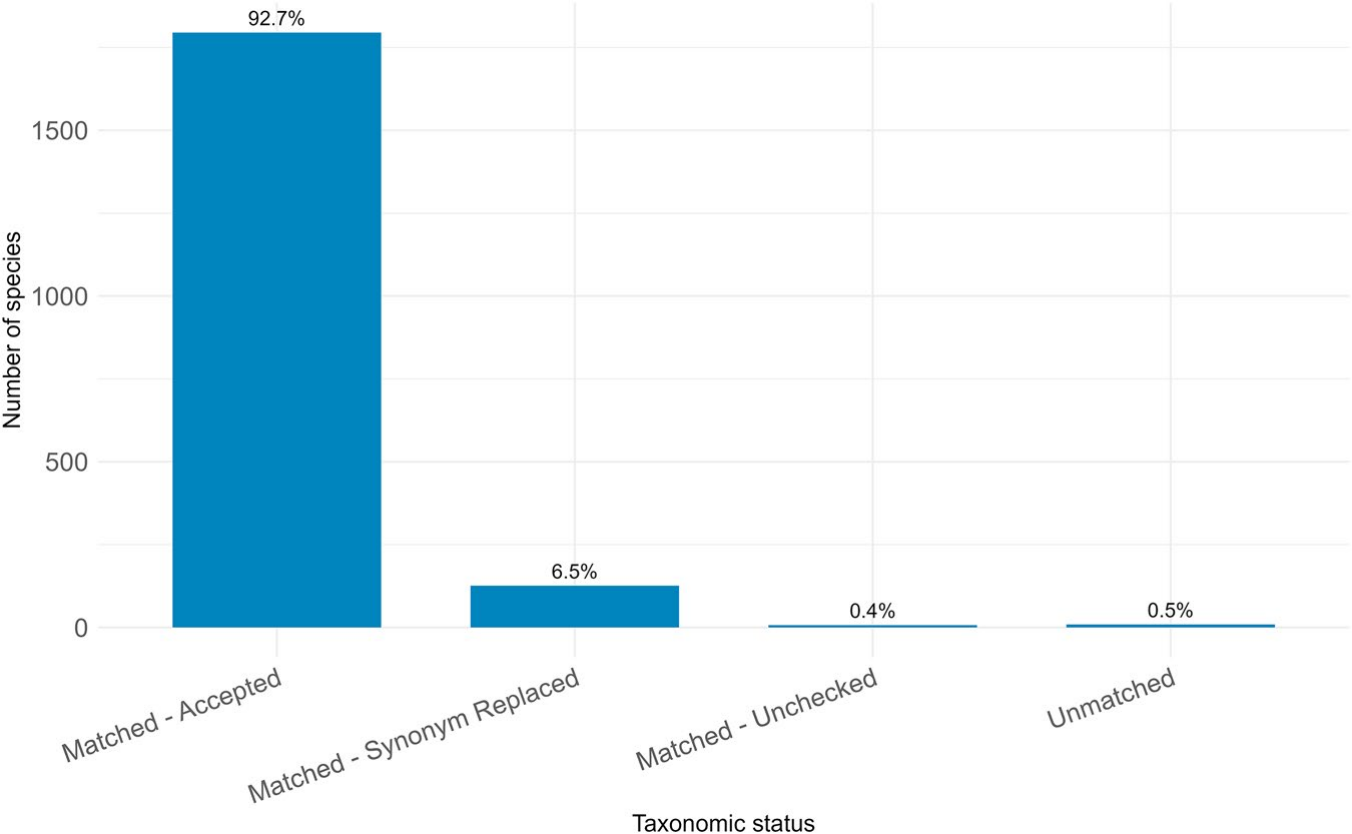
Taxonomic harmonization successfully validated the scientific names for the vast majority (99.5%) of the 1,937 plant species using the World Flora Online (WFO) taxonomic backbone. Most species (92.7%) were already accepted names in WFO, while 6.5% were identified as synonyms and replaced with accepted names. A small proportion (0.4%) were matched with an “Unchecked” taxonomic status, which means that provisional names were found in the backbone pending confirmation or synonymisation. 0.5% of species remained unmatched.

Of the 2,084 collated African plant species, 1,937 were retained following preliminary data cleaning to exclude incomplete or inconsistently entered names and proceeded to taxonomic harmonization. 1928 species names were successfully matched to entries in the WFO database, while nine species remained unmatched. Among the matched names, 1921 were resolved to accepted names: 1795 were already accepted in WFO, while 126 were identified as synonyms and replaced with their corresponding accepted names. An additional seven names had a taxonomic status of “Unchecked,” indicating provisional (uncertain or unresolved) placement in the WFO taxonomy (Fig. 1).

### Classification parameters and PFT mapping

To classify plant species to the JULES PFTs, we required five critical parameters: growth form, leaf type, leaf phenology, photosynthetic pathway, and climatic zone. By systematically identifying these critical classification parameters, we support the wider use of this information to linking trait observations to other PFT taxonomies, supporting parameterization, benchmarking, and ecological modelling efforts across multiple land surface models and plant trait based ecological frameworks^26–28^.

Growth form parameter considers whether each species is a tree, shrub, grass, herb, or fern. For this study, the growth-form parameter records a primary growth form for each species based on published description. The distinction between trees and shrubs for woody plants are based on several interrelated factors such as life-history strategy, height potential, and structural growth patterns which is not always clear-cut^34,35^. For this classification, distinctions between trees and shrubs were based on published species descriptions, and curated database listed in our sources. Photographs from the listed databases were consulted only as corroboration where available. While our classifications align with common functional distinctions relevant to land surface modelling, we recognise that these categories can reflect an ecological continuum rather than strict taxonomic boundaries^36^. Leaf type of plant species describes the morphology of the plant’s leaves, distinguishing between broadleaf and needleleaf types. This is important for photosynthesis efficiency, water use, and plant physiology^37–39^. Leaf phenology involves the seasonal patterns of leaf development, and considers whether the plant is deciduous, evergreen, or semi-deciduous. This classification is important as it reflects adaptive responses to environmental conditions such as water availability, temperature, and light, which in turn influence overall ecosystem productivity and carbon dynamics^40,41^. The photosynthetic pathway is the biochemical mechanism a plant uses for photosynthesis, categorized as C3, C4, and/or CAM pathways^42,43^. The climate zone a species occupies indicates whether it is adapted to tropical or temperate climatic conditions.

Out of the 1,928 species retained after taxonomic harmonization, only 1,465 were found in the existing TRY - Categorical Traits Dataset^44^. TRY - Categorical Traits Dataset is a harmonized summary of categorical trait information for 66,043 plant species including attributes such as the species name, family, genus, as well as four of the critical classification parameters (growth form, leaf type, leaf phenology, and photosynthetic pathway). However, of the 1,465 species found in the existing TRY Categorical Traits Dataset, only 265 (18.1% of the total) were associated with enough information to be mapped to PFT classes. To complete the missing information on key classification parameters (growth form, leaf type, leaf phenology, photosynthetic pathway, and climatic zone) for species with insufficient data in the TRY Categorical Traits Dataset (1,200) and for the additional species not represented in TRY (463), 1,663 in total, we consulted authoritative databases and peer-reviewed literature (Table 2); general web sources (like Wikipedia and Selina Wemucii) were used only to cross-reference incomplete information in authoritative sources.

**Table 2:** Information sources used to identify parameters critical for PFT classification.

Data source	Links
African Plant Database	africanplantdatabase.ch/en
The Floral of Central Africa	www.floredafriquecentrale.be/#/en/home
Useful Tropical Plants	tropical.theferns.info/
World Flora Online	www.worldfloraonline.org/
iNaturalist	www.inaturalist.org/
Global Biodiversity Information Facility	www.gbif.org/
The grass genera of the world	www.delta-intkey.com/grass/index.htm
Selina Wamucii	www.selinawamucii.com/plants/
Wikipedia	www.wikipedia.org/
Publications including^46–67^:	

Following assignment of these parameters, species were assigned to JULES PFTs^10^ using a structured decision flowchart (Fig. 2). It is important to note that some PFT classifications do not require knowledge of all five parameters; for instance, grasses can be classified based on growth form and photosynthetic pathway while woody plants typically required at least three traits (growth form, leaf type, and phenology).

**Fig. 2:**
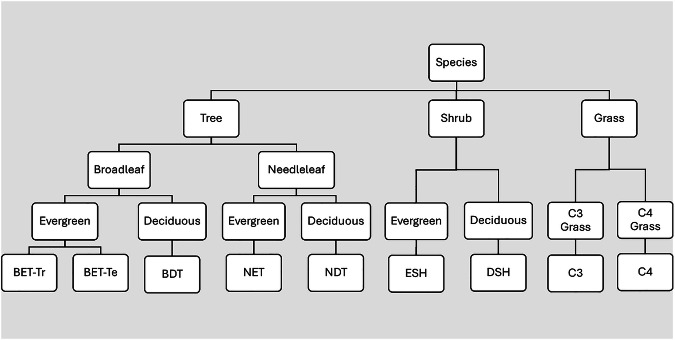
Underrepresented plant species were systematically classified into functional types based on growth form, leaf traits, photosynthetic pathway, and climatic zone to support ecological representation in land surface models.

#### Trees

All tree species were assigned to their own group and further classified based on whether they are broadleaf or needleleaf. Species characterized as having scaled leaves were included in the needleleaf category. The broadleaf trees were further subdivided based on their leaf phenology.

All species that followed the characteristics: tree – broadleaf – deciduous were assigned as the broadleaf deciduous trees (BDT) PFT. Broadleaf evergreen trees (BET) were divided into two subclasses to reflect their ecological and biogeographical distinctions – tropical broadleaf evergreen trees (BET-Tr) and temperate broadleaf evergreen trees (BET-Te). All BET species classified within the tropical subclass were assigned to BET-Tr, and those in the temperate subclass were assigned to BET-Te.

All species that followed the characteristics: tree – needleleaf – deciduous were assigned as needleleaf deciduous trees (NDT) PFT, and tree – needleleaf – evergreen was assigned the needleleaf evergreen trees (NET) PFT.

#### Shrubs

All species classified as either shrub, herb/shrub on the dataset were assigned to the shrub class. This group of species were further separated into two groups based on whether they are evergreen or deciduous. Therefore, all species identified as shrubs/herbs – deciduous were assigned to the deciduous shrubs (DSH) PFT, and those that followed the characteristics: shrubs/herbs – evergreen were assigned to the evergreen shrubs (ESH) PFT.

#### Grasses

All species whose growth form is graminoid were assigned to the grass class. This group was further separated based on their photosynthetic pathway characteristics. Every grass species whose photosynthetic pathway is listed as C4 and C4/CAM were assigned to the C4 grasses (C4) PFT, while all grass species listed as having the C3 and C3/CAM were assigned to the C3 grasses (C3) PFT.

## Data Record

All data and companion materials are deposited on Zenodo^45^ at 10.5281/zenodo.16533069. The deposit contains the following files:

The Mapped_PFT_Harmonized.csv contains the final output of the plant functional type (PFT) mapping and taxonomic harmonization process. This is the final output product.

DATA_column_descriptions.csv provides definitions and descriptions for each column in the dataset. This metadata file serves as a reference to understand the contents and structure of the data.

Traits_observed_from_TRY_Database.csv provides the list of trait observation requested from the TRY database with their accompanying trait ID as in TRY.

TRYdata_analysis.R contains the initial steps for accessing and extracting trait data from the TRY Plant Trait Database. The script makes use of methods and examples adapted from the rtry package, which provides a standardized interface to interact with TRY data.

Workflow_for_PFT_classification.R contains the full processing pipeline for classifying plant species into Plant Functional Types (PFTs). This script brings together the trait data extraction, taxonomic harmonization (based on the World Flora Online approach) and PFT assignment, and generates the final cleaned dataset used for analysis.

Taxonomic_Harmonization_WFO.R contains the script used for taxonomic harmonization of species names using the World Flora Online (WFO) database. This method was used in the final workflow to standardize species names, resolve synonyms, and ensure consistency across plant trait records.

Taxonomic_Harmonization_LCVP.R contains an alternative approach for taxonomic harmonization using the Leipzig Catalogue of Vascular Plants (LCVP) via the lcvplants R package. Although this method was tested, it was not used in the final analysis. The script is retained here as a reference for future comparisons or alternative workflows.

The JULES_pub_count.R script: This script uses the europepmc R package to search for publications related to the JULES land surface model in the Europe PubMed Central database. Keywords were used to retrieve relevant literature for background research and citation tracking.

**Fig. 3:**
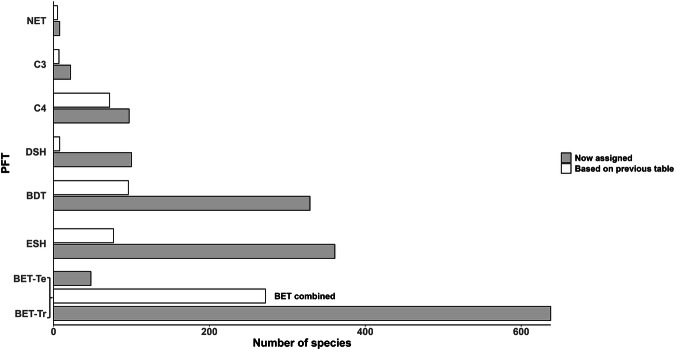
Our assignment of parameters enabling functional classification achieved a sixfold increase in the number of species from the TRY plant traits database that could be linked to the JULES PFT classes, up from 265 to 1,603. The majority of functionally classified African species in this dataset are tropical broadleaf evergreen trees or evergreen shrubs.

Our assignment of parameters enabling functional classification achieved a sixfold increase in the number of species from the TRY plant traits database that could be linked to the JULES PFT classes, up from 265 to 1,603. Figure 3 summarises the number of species assigned to each JULES PFT class in the final dataset as compared with the assignments supported by the most accessible resource previously available (the TRY categorical traits lookup table). Most species were classified as tropical broadleaf evergreen trees or evergreen shrubs, while fewer species were assigned to C3 or C4 grasses or needleleaf evergreen trees, reflecting both ecological composition and gaps in available data. The dataset was compiled in 2025**;** future updates may include new species and/or refinements to parameter thresholds. This sample of 1,603 species from 137 families sampled phylogenetic diversity widely across the continent including the most species-rich clades of flowering plants (including monocots and especially eudicots). However, 325 species remained unclassified mainly because the minimum set of critical parameters required to determine PFT assignment was unavailable from the sources consulted at the time of classification. Also, some important evolutionary lineages are underrepresented (e.g., Gymnosperms, Pteridophytes, Annonaceae, Moraceae, Combretaceae and Sapindaceae).

In terms of trait observations for PFT-level analysis, our data table delivers a fivefold increase in the number of useable observations from 7,373 to 35,537 among the 27 traits that we examined. No physiological or structural trait values (e.g., SLA, wood density) from TRY are included in the dataset. Our new PFT classification table includes original (TRY AccSpeciesID) and harmonised species names, family, growth form, leaf type, phenology, photosynthetic pathway, and climatic zone as well as a resulting PFT classification according to the JULES taxonomy.

## Technical Validation

Commensurate technical validation is incorporated throughout the methodology reported above. Where classification parameters were missing or incomplete in the TRY categorical lookup table, we assigned parameters consistently using cross-referenced information derived from peer-reviewed literature and other online sources (Table 2). The taxonomic harmonization process also served as a technical validation check on the validity of all species records in the dataset. Our codebase and outputs are shared in the Open Research section.

## Usage Notes

These data are intended to enable ecological modelling, functional biogeography, and trait-based vegetation analyses, particularly to redress the underrepresentation of African ecosystems in global vegetation datasets. They are suitable for regional- to global-scale analyses of plant functional diversity, land–atmosphere interactions, or vegetation model parameterisation in Africa and beyond. While our classification aligns with JULES PFT categories, our critical classification parameters can serve as a foundation for linking traits in other models that rely on trait-based plant functional classifications. While we were able to assign critical classification parameters for the majority of species, 15.9% of the species considered could not be assigned to PFTs due to incomplete ecophysiological descriptions, reflecting a wider issue of the understudy of African species in global datasets.

## Data Availability

All data and companion materials are deposited on Zenodo at 10.5281/zenodo.16533069. The archive contains the PFT classification lookup table and supporting files, packaged as a versioned archive and licensed under CC0 1.0. The main lookup table is provided as Mapped_PFT_Harmonized.csv, which contains the finalized PFT classification. This table is intended for direct reuse as a lookup resource when assigning plant species to PFTs in ecological analyses and land-surface modelling workflows. A detailed description of every file and field is provided in README.md and DATA_column_descriptions.csv within the archive. These documents define variable names, codes, and dataset structure to facilitate reproducibility.

## Code availability

The full codebase for the analysis and resulting outputs are available at 10.5281/zenodo.16533069 under CC0 1.0 license.
